# Hospital and Institutional News

**Published:** 1919-01-11

**Authors:** 


### HOSPITAL AND INSTITUTIONAL NEWS.

### K.C.B. FOR GENERALS GOODWIN AND BURTCHAELL

The King has promoted Lt.-Gen. Thomas H. J. C. Chapman Goodwin, C.B., C.M.G., D.S.O., K.H.S., Director-General of the Army Medical Services, to be a Knight Commander of the Bath (Military Division), for services in connection with the war. A similar distinction has been conferred on Maj.-Gen. (Temporary Lt.-Gen.) C. H. Burtchaell, C.B., C.M.G., M.B., K.H.S., Director-General of the Army Medical Services in France, for valuable services rendered in connection with the military operations in France and Flanders. The honours conferred upon Lieut.-Generals H. J. C. Goodwin and C. H. Burtchaell will be received with supreme satisfaction by the medical profession and by everyone who has been in touch with their careers and work. They had to take up grave responsibilities and to face innumerable problems which called for the exercise of wisdom, judgment, courage and administrative gifts of the highest, when they assumed office as successors to Sir Alfred Keogh and Sir Arthur Sloggett respectively. The result has won for them golden opinions from all who have been brought into close association with them in the discharge of their onerous and responsible duties. The honours conferred upon them by the King have been welcomed by the profession and the Army, and their success and devotion afford once more proof of the wisdom, which has characterised the selection and appointments to great offices connected with the Army, which have resulted, in God's good providence, in the triumph of our forces and the defeat of the enemy. The following honours are also announced for valuable services rendered in connection with the war: *K.C.M.G.*, Maj.-Gen. William Watson Pike, C.M.G., D.S.O., F.R.C.S.I., Army Medical Service; Temporary Colonel John Atkins, C.M.G., M.B., F.R.C.S., Army Medical Service: *K.B.E.* (Military Division), Temp. Hon. Col. John Lynn Thomas, C.B., C.M.G., F.R.C.S., Col. Harry Edwin Bruce-Porter, C.M.G., Army Medical Service (T.F.).

### SIR LYNN THOMAS AND LIMBLESS WARRIORS.

The honour conferred upon Colonel J. Lynn Thomas, C.B., C.M.G., F.R.C.S., referred to in the preceding paragraph, must undoubtedly be due largely to his excellent work during the past two years in connection with the establishment of the Prince of Wales' Hospital for Limbless Sailors and Soldiers at Cardiff. In 1916 it became evident that other fitting hospitals must be started to relieve Roehampton, and the suggestion was put forward that one should be opened at Cardiff to deal with limbless men resident in Wales and Monmouthshire. Within a few days after the original suggestion Colonel Lynn Thomas had a scheme on foot and with the aid of two generous Cardiff Docksmen (Mr. W. Percy Miles and Mr. J. P. Cadogan) had arranged to purchase the old Mansion and two adjoining houses. The sites of these houses cover an area of three quarters of an acre and it has been possible to complete a scheme for dealing with the fitting of limbless men, which will bear comparison with any other fitting hospitals in Great Britain and Ireland. In fact, several features introduced by Colonel Lynn Thomas are now being adopted by other fitting hospitals, and he has undoubtedly stamped his personality upon the whole scheme. During the war Colonel Lynn Thomas has also acted as Assistant Inspector of Military Orthopædics in the Western Command and the new honour will be much appreciated by his friends in the Principality, in view of his arduous work during the past four years. Colonel Lynn Thomas was Senior Surgeon for the Welsh Hospital in the South African War and in recognition was made a C.B., and a few years ago the C.M.G. was also conferred upon him. He is a Justice of the Peace for Cardiganshire and Glamorgan, and was High Sheriff of Cardiganshire in 1907-8.

### A KING EDWARD'S FUND FOR GLASGOW?

The great Glasgow hospitals have so long been in debt that it is refreshing to learn of a big scheme for lightening their liabilities. A correspondent in the *Herald* has proposed an endowment fund of £2,000,000, to provide an income of £100,000. This would be a partial endowment, and might perhaps be more practical than an attempt to endow the three great hospitals severally. A kind of King Edward's Fund for the Glasgow hospitals might be formed. At all events, that Fund remains an example of what can be done and how it can be conducted. Its success shows that the old argument that it is a "rival" to the general hospitals is fallacious; and the present attempt, if carried out, would be a war memorial not unworthy of the city.

### TWO GIFTS TO CARNARVON.

Mr. Owen Jones, who has entrusted Sir W. Goscambe John with the task of carving a statue of Mr. Lloyd George for Carnarvon, has also decided to add a children's wing to the Carnarvon Cottage Hospital.

### A CHILDREN'S HOSPITAL FOR ILFORD.

The Ilford Maternity Home and Children's Hospital Committee has suggested to the Ilford Urban District Council that its War Memorial should be a Children's Hospital.

### A NEW INFIRMARY FOR BANGOR?

It has been decided to raise the necessary sum of money to build a new infirmary at Bangor in place of the present Carnarvonshire and Anglesey Infirmary. Mr. E. O. Pryce believes that the money can be raised, and the inadequacy of the present building is generally admitted.

### BETTER ROOMS FOR NURSES.

According to the published list of awards of the King Edward's Hospital Fund for London, the

Great Northern Central Hospital is to receive £6,000, £1,000 of which is in reduction of debt, and £1,000 towards the building of the nurses' home. We are glad to see that the King's Fund has recognised the great need which exists at the hospital for better accommodation for nurses, and everyone will agree that the nurses deserve all that can be done for them in recognition of their services during the war. The extension of the hospital, which has been proposed as a war memorial, includes the erection of a nurses' home, the site for which the hospital already holds. A Christmas appeal for £5,000 was issued. The outbreak of war showed the reserves of charity in this country. Unless the return of peace is similarly welcomed, the change of heart, of which many have boasted, will not have taken place.

### THE DEFEAT OF CAPTAIN HADEN GUEST, R.A.M C.

Captain Haden Guest, R.A.M.C., suffered defeat at the Parliamentary election in Central Southwark, where he was standing as a Labour candidate. He polled 3,126 votes, as against 8,060 given to his opponent, Mr. J. D. Gilbert, the retiring member, who was selected as the Coalition candidate, and has all his life been connected with the division, not only representing it in the House of Commons, but also on the London County Council for a great many years. Captain Haden Guest had none of these advantages. Apart from his work as a medical officer of the London County Council and his clinical work in the borough, he was practically unknown in the constituency until he began to woo it for Parliamentary honours. The result, therefore, cannot be very disappointing to him, although from the medical standpoint his loss to the House must be deplored. Captain Haden Guest is a good speaker, and has a wide and practical knowledge of the varied problems of life, which would have stood the country in good stead had he been sent to the House of Commons.

### PHARMACIST IN PARLIAMENT.

In the recent Parliamentary election only one of the three candidates officially supported by the Pharmaceutical Society of Great Britain was successful. One candidate withdrew at the request of the party Whips, and the Labour candidate was defeated. Mr. W. J. U. Woolcock, the successful candidate, who was elected unopposed to represent Central Hackney, is well known to the general body of Pharmacists and many hospital officers, having been early in his career an assistant Pharmacist at University College Hospital, London. Subsequently Mr. Woodcock was Local Association Officer and the secretary and registrar of the Pharmaceutical Society, vacating this position last year to become manager of the Association of Manufacturing Chemists. In problems concerning Pharmacy Mr. Woolcock is qualified to keep a watchful eye on the interests of the profession. It is perhaps not generally known that Sir Richard Winfrey, secretary to the Board of Agriculture, is a Pharmacist, and though not practising his profession he retains an interest in it.

### AN INFIRMARY SUPERINTENDENT'S SALARY.

The Bristol Board of Guardians has decided to increase the salary of Dr. R. H. Norgate, medical officer of the Stapleton and Eastville Institutions and Children's Homes, from £350 to £450 per annum, with a war bonus of 10 per cent. This increase has been recommended and approved without formal application by Dr. Norgate, out of regard for "the importance and quantity" of his work.

### CLOSING OF MILITARY HOSPITALS.

One by one the buildings which were adapted to hospitals for the duration of the war are being closed. Those military hospitals which were few and small, and supported by voluntary funds, have been among the first to cease operations. The winding up has had humours as well as difficulties. Equipped largely by loans of furniture, beds, and bedding, the original donors, when invited to claim their property, include some who complain that the sofa which they lent has been returned without its casters, that the linen has suffered in the wash, and that the piano shows signs of wear and tear. But the cost of closing is not only found in a ruffling of temper. Buildings have suffered too, and though the temporary hospital agreed to bear the cost of repairs, the income which was so constant while the hospital was full often ceases immediately. So that these hospitals, which have always paid their way, may close with a debt of, say, £300, for which a final appeal has to be made.

### THE SLUMS AND SMALL LANDLORDS.

Like other cities, Bradford is faced with the problem of housing; at least 10,000 new houses will have to be built. Bradford also possesses in the Chairman of its Health Committee a gentleman who is accustomed to press his views upon the City Council. He has published a collection of his speeches, together with a reply to a criticism they received. In brief, Mr. E. J. Smith, the writer, favours the urbanisation of the country by building the new houses in its outskirts, and connecting them by trams and trains with the city itself. This process in time, he hopes, will lead to the clearing of the slums in the centre. In his own words, a "scheme for the self-contained villages to be erected on the hilltops surrounding the city has been proposed, with the suggestion that each should contain not only 1,000 houses of the bungalow type, but also communal cooking kitchens, laundries, hot-water service, baths, schools, libraries, isolation rooms, playing fields, allotment gardens, churches, Sunday schools, and the service by trams run at a universal penny fare." Perhaps the most practical pages are those (19-22) in which the slum problem is discussed, and the difficulties with which municipal housing schemes are beset are described from practical experience. The attraction of house property for the impecunious investor is also noted, and the evil effect of this in maintaining slums is well analysed. Messrs. P. S. King have published the address for 1s., under the title of "Housing: The Present Opportunity."

### "EATING WITH THE DEAD."

Mr. W. Spooner, a member of the Brampton Board of Guardians, in Cumberland, startled his colleagues by a series of revelations in regard to the influenza epidemic, which he advanced to support his view that the closing of the isolation wards at the local workhouse, apparently used once only in forty years, had occasioned many deaths. Mr. Spooner declared that during the influenza epidemic in one family of ten, of whom three died, the rest slept and ate with the dead, and that in another a corpse was lying in the kitchen for four days, with the inmates eating in the apartment. In a third case he declared that a man, his wife, and child had to sleep in the same room as their daughter after her death until the body was removed from the house. The child had since died. In reply it was said that the isolation wards could be used only for those in receipt of parish relief. "Pull them down, then," retorted Mr. Spooner. It would seem that the short staffs of undertakers' firms and public mortuaries explain a condition that reminds us of the tales of Harrison Ainsworth.

### UNFORTUNATE ALLUSIONS.

The Christmas magazine of Queen Alexandra's Hospital for Officers, Highgate, apart from the letter from the Queen Mother with which it opens, is chiefly a history. No contributor seems quite to have the observation of the editor, who is to be thanked for recording a little pastime of the patients which arose from the custom that allowed one of them to choose the Sunday evening hymn. "Here we suffer grief and pain" was not allowed. But one Sunday evening when there was a full moon, after the hymn "We thank Thee for the silver moon by night," the Amen gave the signal for an air raid. That was a coincidence, but in connection with it should be recorded the report current at the time that at the memorial service for the children who were victims of an air raid at a coast town the congregation sang "There's a friend for little children above the bright blue sky." Since such a hymn could have been chosen only by one who tried to be too apt in finding a word for every occasion it deserves to be placed on record as a warning. How few are they who can be trusted with an inscription, a quotation for an occasion, or an epitaph!

### THE FEES OF CONVALESCENT HOMES.

The cost of maintenance in convalescent homes has risen, as in other institutions, though less has been heard of their difficulties. The committee of the Moseley Hall Convalescent Home for Children, Birmingham, has therefore decided to reduce from two weeks to one week the period secured by a half-guinea ticket of admission, though the stay of each patient shall be a fortnight, as before. The Children's Hospital is to pay 5s. a week instead of half-a-crown per child, and all patients who come from other institutions will have to pay 7s. 6d., instead of the old fee of 5s. The Children's Hospital has agreed to this change of fees.

### NEW USE FOR LONDON "DAILIES."

Mr. R. J. Coles, secretary of King's College Hospital, in a brief letter to the newspapers, has asked for the gift of a diathering apparatus for the hospital's operation theatre. This direct request has a personal confidence and directness which makes it noteworthy. The London dailies are rarely used for small, direct appeals. We hope to hear from Mr. Coles that his attempt has been already successful.

### EX-SERVICE MEN AND CERTIFICATES.

Considerable difficulty has been experienced by the War Pensions Committees in London in obtaining certificates with regard to men discharged from the Forces suffering from tuberculosis. Hitherto these have been given by the ordinary medical referees, but there have been cases when men have had to seek the assistance of a public institution, and the chief medical officer has either declined to grant the necessary certificate or raised difficulties. With a view to removing these difficulties the Ministry of Pensions considers it desirable that the tuberculosis officers in the various districts should give the certificates after examining the men, and the Local Government Board raises no objection to this being done. A fee of five shillings for each certificate is to be paid to the tuberculosis officer. At Lambeth Borough Council when this question was discussed, and the necessary permission was granted to the tuberculosis officer to give the certificates, Councillor Dr. J. McKeith, M.B., C.M., drew attention to the inadequacy of the fee, and said from his own experience the examination could not be done for the amount. It was pointed out that doctors were doing what was required for a fee of half-a-crown, with a view of helping discharged warriors in the circumstances indicated.

### MEMORIAL TO A MEMBER OF THE STAFF OF THE SATURDAY FUND.

Sir T. Vezey Strong, chairman of the Hospital Saturday Fund Executive Committee, has unveiled a photograph of Lance-Corporal Christopher William Axworthy, who was a member of the staff at the head office until he volunteered in June 1915. He joined the 11th Battalion of the Royal Fusiliers and was killed last September by a German sniper when attending a wounded man. Thirteen members of the staff joined the Army.

### BRIGHTON'S NEGLECTED HOSPITAL BOXES.

The Hospital Sunday collection in Brighton, Hove, and Preston shows a drop of just over £300 on the sum raised in 1917, but it must be borne in mind that, unfortunately for the medical charities, the previous Saturday was a flag day, and, moreover, the Sunday was one of the wettest on record. But there is a notable and unsatisfactory feature in the balance-sheet just published, and that is the result of the collections in the hospital-boxes at the hotels. Twenty such institutions subscribed in their boxes less than £20, one contributing the magnificent sum of 4½d.! It shall be nameless. Another—one of the largest in Brighton—gave 2s. 10d. A little effort on the part of those entrusted with hospital boxes would surely ensure the collection of a larger sum than £18 10s. 7d. in twenty hotels in twelve months, and Brighton was never so full of visitors as this.

### AN ELEVENTH HOUR WARNING.

The now admitted need for numerous more hospital beds is being enforced by the situation created through the influenza epidemic, during which cases nursed at home apparently showed a higher rate of mortality than those treated in hospital, and by the proposed Ministry of Health. That official will have not only to enforce preventive measures but to supply sufficient accommodation for the sick which will occur. Happy is that hospital which possesses sufficient beds without the help, and the accompanying conditions, of the future Ministry. The virtue of the voluntary system, as of other good and ancient things, are apt to be appreciated most when the system itself has been displaced by centralisation under the specious name of efficiency. So we hope that the voluntary hospitals and their supporters will raise the necessary funds for the urgently-needed extensions before the doubtful aid of the State is imposed upon them: *timemus Danaos et dona ferentes.*

### "DURATION" OF WAR HOSPITALS.

Recently the Lewisham Guardians complained that it was difficult to secure nurses for the war hospital because candidates and themselves were uncertain how long the place would be open. Inquiry was accordingly made at the War Office, which now replies: "The position as regards military patients is not as yet sufficiently stabilised to permit of anything approaching an accurate estimate being made in the matter of the user of the Lewisham Military Hospital. It would perhaps be only misleading to your Board to say more than that it is anticipated the institution will be required for a further period of from three to six months."

### BATTERSEA GENERAL HOSPITAL SCHEME.

Before the outbreak of war a contract was entered into by the Board of Management of the Battersea General Hospital (Incorporated) for the erection of a new and thoroughly up-to-date out-patients' department and this has, despite building difficulties, been completed, except as regards certain necessary equipment. The whole structural cost and architect's fees, etc., have been settled, but a sum of £1,000, at least, is required before the building can be opened. The Board regard the old arrangement for out-patients, which is still in use, as very far from satisfactory, and are anxious to be in a position to open the new and convenient buildings without much further delay.

### A MYSTERIOUS DISEASE

A new regulation concerning acute encephalitis lethargica and acute polio-encephalitis has been made by the Local Government Board in order to secure the notification of an obscure infectious disease which occurred in epidemic form last spring. The cases presented a group of unusual cerebral symptoms, some of which were said to resemble those of a rare disease called botulism, which is due to the consumption of infected food. The illness presented the character of an acute general disease associated with progressive languor, apathy, and drowsiness, passing into lethargy, muscular weakness passing into complete disablement, and paralysis of various muscles, chiefly of the eyes and face. The outbreak gave rise to considerable anxiety, as the course, duration, and fatality of cases were serious, and their nature was obscure. Investigation proved that the disease was not botulism, but that its clinical manifestations appear to constitute, on the balance of evidence, an infectious disease unrecognised until recently, and probably distinct from the form of acute poliomyelitis which attacks the brain. Hence the new notification, which the Local Government Board explains to medical officers of health, is important in order to secure early and complete knowledge of all cases, which should be carefully studied in close consultation and collaboration with the doctors in attendance.

### A THEME FOR THOMAS HARDY.

When the military took over the Stairbridge Workhouse for a hospital two inmates each named Edward Evans were transferred to Kidderminster Workhouse. One, who came from Walsall, was transferred later to Worcester Union, but eventually returned to Walsall Union; the other, who came from Birmingham remained at Kidderminster where he lately died. By an accident the Walsall man's relatives were informed of the death. So they attended the deceased's funeral and procured two certificates, and the supposed widow visited his grave. Then the mistake was discovered, whereupon the son of Walsall Evans claimed his expenses, his own and his mother's railway fare, his loss of a day's work, and the cost of the two incorrect death certificates. The matter is being considered, but whether the dead man had any relatives who now have to mourn for him does not appear. If so here is another of life's little ironies for Mr. Hardy.

### THIS WEEK'S DRUG MARKET.

Business continues very slow, and in the absence of business the downward movement in prices is still in evidence. The dulness of the demand can no longer be attributed to the holiday season or to stock-taking, as both of these are well over. The main reason is that buyers are acting with extreme caution and are not increasing their stocks, their hope being that they will be able to carry on with existing stocks until prices have further receded. Several of the synthetic drugs are quoted at lower rates; this applies to antifebrin, aspirin, benzoic acid, phenacetin, and salol. On the other hand, the high value of phenazone is maintained. The demand for eucalyptus oil is not so keen, and the price tendency is lower. Clove oil is cheaper and lemon oil tends downwards in price. There are prospects that potassium permanganate will soon be much cheaper, and it is also possible that sugar of milk will shortly be procurable at lower rates. Glycerophosphates and lithia salts have a tendency to advance in price.

